# Restriction of Access to Healthcare and Discrimination of Individuals of Sexual and Gender Minority: An Analysis of Judgments of the European Court of Human Rights from an Ethical Perspective

**DOI:** 10.3390/ijerph19052650

**Published:** 2022-02-24

**Authors:** Tobias Skuban, Marcin Orzechowski, Florian Steger

**Affiliations:** 1 Kbo-Isar-Amper-Klinikum Region München, 85540 Haar, Germany; tobias.skuban-eiseler@kbo.de; 2 Institute of the History, Philosophy and Ethics of Medicine, Ulm University, 89073 Ulm, Germany; florian.steger@uni-ulm.de

**Keywords:** access to healthcare, discrimination, LGBTQI, ethics, international law, vulnerable population

## Abstract

Individuals of sexual and gender minority (SGM) form a vulnerable group with specific healthcare needs that might be prone to experience discrimination and restrictions regarding their access to healthcare. As the judgments of the European Court of Human Rights (ECtHR) offer a normative perspective on these issues, we analyzed them systematically (1) to identify whether and in what manner ECtHR’s judgments concern restriction of access to healthcare for SGM individuals and (2) to identify and categorize the ways of discrimination to which SGM individuals are exposed. We conducted a systematic search of the database of the ECtHR’s judgments with the use of specified search terms. Descriptive statistics were performed on the identified judgments. Subsequently, we analyzed the judgments with the use of a qualitative method of thematic analysis. We identified n = 73 cases relevant for our study. In n = 7 (9.59%) of judgments, we found limitations of access to healthcare for SGM individuals, e.g., in cases of restrictions for transsexual individuals to receive hormone or surgical therapy. We regard this as a specific form of discrimination. Furthermore, we identified five other categories of discrimination: restriction of parental rights, failure to respect one’s gender identity/sexual orientation, discrimination by jurisdiction, prohibition of promotion, and verbal/physical attacks. The ECtHR proves to have a balanced view on the sensitive topic of sexual self-determination condemning any form of discrimination or restriction of access to healthcare. However, there is a need for further research on discriminatory acts by other individuals, e.g., healthcare providers, rather than by public authorities.

## 1. Introduction

Although there has been considerable progress regarding the acceptance and protection of gay, lesbian, bisexual, transgender, and non-binary gender individuals (hereinafter ‘individuals of sexual and gender minority’, SGM), they still suffer from substantial discrimination and disparities regarding their access to healthcare [1,2], especially in the case of transsexual persons and their access to transition-related care [3]. SGM individuals also report experiences of discrimination by healthcare providers [4,5], who often lack specific and adequate training concerning the needs of SGM individuals [6,7,8]. Although higher levels of knowledge regarding SGM individuals have been found to be correlated with more positive attitudes towards SGM persons [9], individual cases of discrimination in healthcare can be based on healthcare professionals’ moral convictions and attitudes. Other barriers include high costs, e.g., for the specific interventions transsexual individuals need, lack of availability of specific health services, former negative experiences in healthcare settings, prejudice amongst healthcare professionals, unfavorable communication between healthcare providers and patients, discrimination and stigmatization, and insurance-related issues such as the unwillingness of insurance companies to pay for transgender-related care or gaps in healthcare insurance coverages [6,10,11,12,13,14,15,16]. There is a considerable systemic aspect to discrimination and the restriction of access to healthcare. Some areas, such as rural regions, seem to be prone to more frequent acts of discrimination. This can occur through the development of a specific atmosphere of discrimination and results in a phenomenon of ‘health migration’, e.g., transsexual individuals changing their place of residence in order to get better access to trans-specific healthcare [17,18,19,20,21].

Barriers in access to healthcare are an example of a specific form of inequity; however, SGM individuals also experience other discriminatory practices. The EU-LGBT II survey of 2019, conducted with 139,799 persons in all EU Member States and the candidate states of North Macedonia and Serbia showed an alarming degree of individuals that experienced harassment in form of threatening or offensive situations (58%). Over half of the respondents stated that they are almost never or rarely open about having a different sexual orientation or gender identity, 33% keep away from certain places for fear of being assaulted, threatened, or harassed. Only 11% of the respondents reported the most recent incident of discrimination they experienced to the authorities as they believed that nothing would really change [22].

This discrimination and these restrictions regarding access to healthcare for SGM individuals lead to an increased risk to suffer from physical and/or mental diseases [23]. Minority stress by experiences of discrimination and victimization leads to higher rates of anxiety, depression, drug abuse, self-harm, and suicidality and to multiple physical effects such as a negative impact on the cardiovascular, metabolic, and hormonal system [23,24,25]. In this context, it should be stated that the question of gender identity and sexual orientation has been traditionally not only a topic pertaining to healthcare but also a legal issue. The legal system determines the framework for medicine to provide certain treatments or not, to make healthcare accessible for everyone, and to prevent discrimination. In so doing, the legal system can provide a climate that is prone to discrimination of minority groups such as SGM individuals. As an example, Russia with its legal barriers to freedom of expression on sexual and gender diversity issues [26]. Abandoning anti-SGM laws abetting discrimination and restrictions of access to healthcare is crucial to eliminate minority stress and hence related negative health impacts for SGM individuals.

In this paper, we seek to elucidate ethical and legal discourses concerning the discrimination and restriction of adequate access to healthcare for SGM individuals. Through systematic assessment and categorization of all judgments of the European Court of Human Rights (ECtHR) concerning SGM individuals, we aim to better understand and illustrate medico-ethical and legal discourses on human rights violations of SGM persons. The judgments of the ECtHR provide not only legal considerations but also normative assessments regarding central ethical questions. They give orientation, are aimed to provide legal certainty, and point to violations of human rights. The jurisdiction of the ECtHR should be binding for all 47 Member States of the Council of Europe; however, some states refuse to acknowledge particular judgments. Before the ECtHR proceeds an application, the case needs to pass through the proceedings at the national level, i.e., it requires a decision of national courts and the highest domestic courts. Only after this process, the application can be submitted to the ECtHR. This means that cases brought before the ECtHR have usually already been ruled on by national courts and represent only the tip of an iceberg. The case is being proceeded by the ECtHR if it satisfies certain eligibility criteria: exhaustion of domestic remedies, 4-month deadline from the domestic judicial decision, complaint relevant to the Articles of the European Convention of Human Rights (hereinafter ‘the Convention’), and significant disadvantage of the applicant. After examination of admissibility and merits of the application, the ECtHR judges on violation or non-violation of the Convention [27].

The judgments of the ECtHR provide an interpretation of the Convention. The Convention is an internationally binding agreement guaranteeing fundamental human rights and freedoms in the Member States of the Council of Europe. The Convention consists of three sections. Section I (Articles 2 to 18) contains Articles on main rights and freedoms, e.g., Article 2 “The right to life”, Article 3 “Prohibition of torture and cruel, inhuman and degrading treatment”, Article 14 “Prohibition of discrimination”. Section II (Articles 19 to 51) defines the setup, tasks, and rules of operation of the ECtHR. Section III (Articles 52 to 59) contains miscellaneous concluding provisions. Moreover, the Convention contains 15 additional protocols amending the framework of the convention system or expanding the rights that can be protected. As of 2010, these protocols are open for signature and ratification by the Member States—with some states opting out from signing particular protocols.

The human rights protection as exercised by the ECtHR operates on two levels: substantive and procedural. The substantive aspect involves protection of basic rights and criminalization of a conduct endangering human rights. It is the obligation a government has to its people. At the procedural level, the human rights law protection implies an obligation of a state for an action through an available and effective procedure, i.e., an obligation to investigate, prosecute and, if appropriate, punish human rights offenses.

The judgments of the ECtHR have an impact on the legal situation of SGM individuals, and, indirectly, on the health of this group through decreasing mental stress in certain situations. One prominent example concerns the decriminalization of homosexuality in the Republic of Cyprus. Originally retained from the United Kingdom Criminal Law Amendment Act of 1885, Articles 171–173 of the Criminal Code effectively criminalized homosexuality and sexual acts between consenting males [28]. Following a ruling of 22 April 1993, in which the ECtHR deemed this a violation of the Convention, the Parliament of the Republic of Cyprus was forced to change this law and to decriminalize same-sex sexual contacts on 21 May 1998. In 2002, the Parliament equalized the age of consent for heterosexual and homosexual individuals to 17. Similarly, decriminalization of homosexuality in the area under the Turkish Cypriot administration in the north of the island was only achieved in 2014.

In our research, we address the following questions: (i) How many ECtHR judgments are concerned with the healthcare of SGM individuals? (ii) How can these judgments be thematically grouped regarding various types of discrimination and restricted access to healthcare? (iii) How does the Court assess these cases, especially regarding their ethical implications?

## 2. Materials and Methods

Analyzed judgments of the ECtHR were retrieved from the database HUDOC on 4 February 2022. HUDOC (accessible under: https://hudoc.echr.coe.int/eng#{%22documentcollectionid2%22:[%22GRANDCHAMBER%22,%22CHAMBER%22]}) is a database of the case law of the Court. It encompasses judgments starting from 14.11.1960. Following search terms in English were used: “transgender” and “health”, “transsexual” and “health”, “homosexual” and “health”, “bisexual” and “health”, “lesbian” and “health”, “gay” and “health”, “queer” and “health”, “same-sex” and “health”, “sexual minority” and “health”, “gender minority” and “health”.

**Figure 1:**
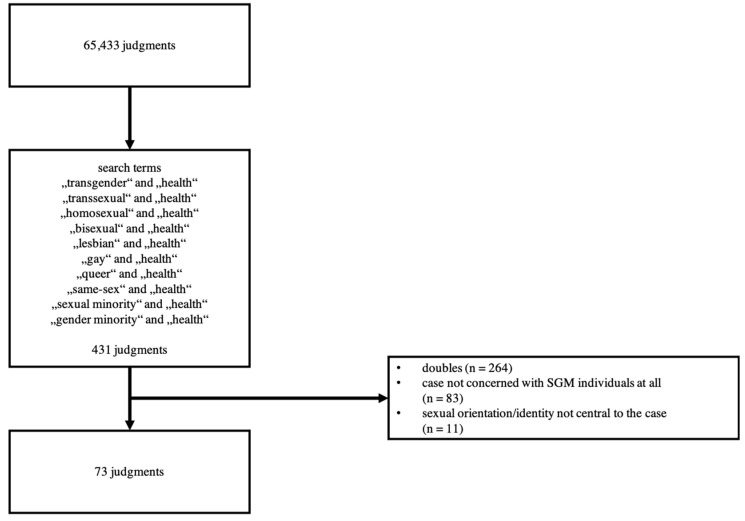
Flowchart of the search.

The search yielded 431 results. After the elimination of duplicates, 167 remaining judgments were read. In total, 73 judgments proved to be relevant for our investigation. 94 judgments that involved cases that were either not concerned with SGM individuals at all or in which sexual orientation or sexual identity was not central to the case were excluded (Figure 1).

### 2.1. Descriptive Statistics

Descriptive statistics were performed on the Articles of the Convention involved in the judgments. All articles which the ECtHR ruled on as well as violations of these articles were counted. The judgments of the ECtHR often involve different articles in one case or even different paragraphs of the same article. For example, a judgment could state that the substantive aspect of an article was violated while the procedural aspect was not. For matters of clarity, we counted a violation of an article if at least one violation of one of its aspects or sub-paragraphs was held by the ECtHR. This constitutes a certain limitation of our investigation.

### 2.2. Thematic Analysis

A thematic analysis was performed on all analyzed judgments. Thematic analysis is a quantitative approach for the identification of recurring themes or patterns in narrative or text materials [29,30]. All 73 judgments that have been found to be relevant for our study have been read and analyzed in their entirety. Based on the analysis of the textual content of the judgments, thematic categories were inductively formulated and critically discussed in the multi-professional team of the authors, including a psychiatrist (T.S.), a physician and expert in the history, philosophy and ethics of medicine (F.S.), and a political scientist (M.O.). These categories represent important thematic patterns of the judgments with regard to the research aim and research questions and do not depend exclusively on quantifiable measurements. A thematic analysis of the full text of documents pertinent to the research question was conducted. Specific statements from the documents were coded manually and thoroughly discussed, in order to identify the data and determine possible categories and sub-categories. Each of the categories is illustrated by one or more reports on the case under deliberation.

## 3. Results

### 3.1. Countries

**Figure 2:**
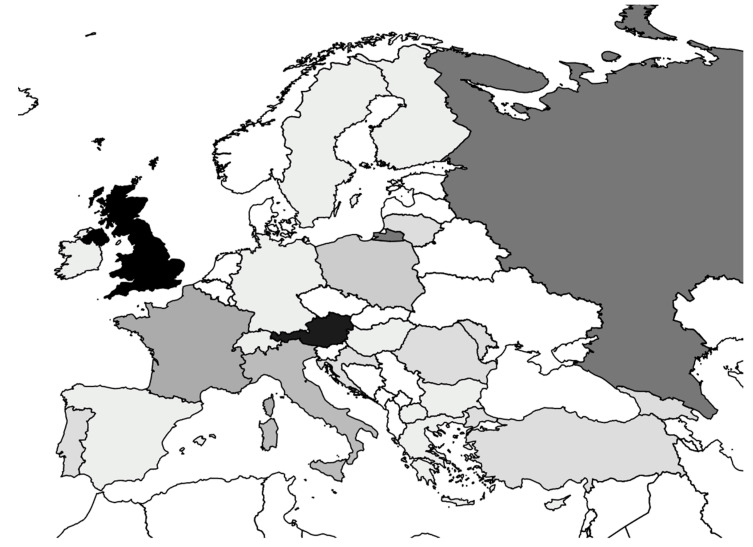
Overview of the states the analyzed cases arise from; the darker the state is colored, the more cases deriving from this state have been found.

The n = 73 judgments involved applications against n = 23 countries. Most applications were filed against the United Kingdom (n = 15), following by Austria (n = 13), Russia (n = 8), France (n = 5), Italy (n = 4) and Poland (n = 3). N = 2 applications derived from Croatia, Georgia, Lithuania, Moldova, Portugal, Romania and Turkey, one application from Bulgaria, Cyprus, Finland, Germany, Greece, Hungary, Ireland, Macedonia, Spain, Sweden, and Switzerland (Figure 2).

### 3.2. Articles of the European Convention on Human Rights

In Table 1 we present frequencies of the Articles of the Convention involved in the analyzed judgments. As articles that are discussed in a judgment may not be found to be violated, we separately present the number of judgments that involve a certain article in the third column, and the number of judgments that find the respective article breached in column four. Most of the judgments involved Article 14 (prohibition of discrimination) and 8 (right to respect for private and family life).

Table 2 gives an overview of the number of relevant judgments we found and the number of articles that have been found to be violated for each country.

### 3.3. Categories

**Figure 3:**
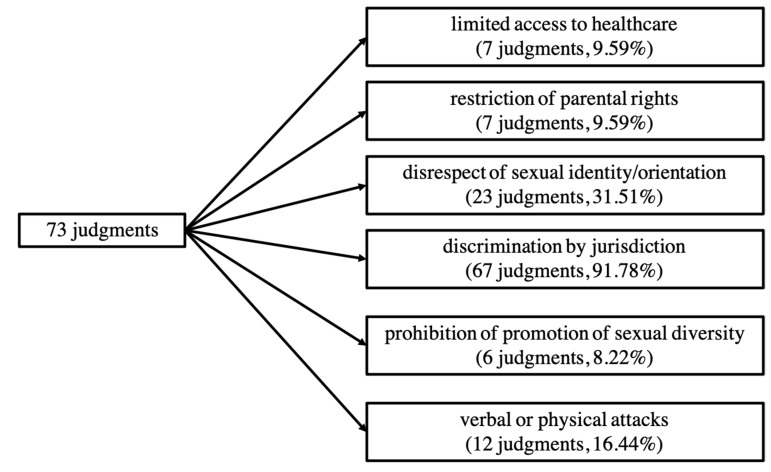
General count and percentages of judgments we found regarding each category.

We formed six partially overlapping categories representing thematic patterns in the analyzed judgments (Figure 3). The categories are not mutually exclusive—often judgments were classified as belonging to several categories. With concern to limitation of access to healthcare for SGM individuals, we found n = 7 judgments (9.59%) that could be classified in this category. We consider limitation of access to healthcare as a form of discrimination. Five further thematic patterns involve other categories of discrimination: discrimination by restriction of parental rights (n = 7 judgments, 9.59%), discrimination by the disrespect of sexual identity/orientation (n = 23 judgments, 32.51%), discrimination by jurisdiction (n = 67 judgments, 91.78%), discrimination by the prohibition of promotion of sexual diversity (n = 6 judgments, 8.22%), and discrimination by verbal or physical attacks (n = 12 judgments, 16.44%).

#### 3.3.1. Judgments Involving Limited Access to Healthcare

We identified n = 7 judgments (9.59%) dealing with restricted access to healthcare. All of these judgments concerned transsexual individuals and their need for specific medical interventions during their transition. One of the cases dealt with a restricted reimbursement of hormone therapy and/or gender reassignment surgery (i), three cases with difficulty to receive hormone therapy and/or gender reassignment surgery due to legal uncertainties (ii) and one case concerning the denial of artificial insemination asked for by a couple of which the male partner was transsexual (X, Y and Z v. the United Kingdom, appl. no. 21830/93). One case concerned the denial of an extension of one partner’s health insurance coverage to the other partner in a male homosexual relationship (P.B. and J.S. v. Austria, appl. no. 18984/02). One case concerned a participant of a Gay Pride parade, who was arrested, physically ill-treated by the police, and not allowed to get in touch with a doctor afterward (Boris Kostadinov v. Bulgaria, appl. no. 61701/11).

Example of (i): The applicant in the case Van Kück v. Germany (appl. no. 35968/97) was a male-to-female transsexual claiming reimbursement for hormone therapy and gender reassignment surgery by her health insurance. The authorities claimed she was not entitled to reimbursement for her treatment. In their argumentation, hormone therapy and surgery were (a) not the only possible treatments for transsexualism and (b) the applicant had deliberately caused her transsexuality by initially taking hormones without first consulting a medical practitioner. The ECtHR saw a violation of Article 6 and Article 8 of the Convention. The Court reiterated that a medical necessity was not a matter of legal definition but a broad term that also can be seen as fulfilled if the psychosocial situation of the applicant can be improved by the medical intervention in question. Furthermore, the Court noted that gender identification is part of the applicant’s self-determination and that this form of self-determination has been violated by the proceedings.

Example of (ii): The applicant of the case L. v. Lithuania (appl. no. 27527/03) was a female-to-male transsexual seeking the medical interventions necessary for gender reassignment. During the treatment, his general physician refused a further prescription of hormones due to a lack of legal certainty regarding gender reassignment surgery, which would have been the next step during the transition. The ECtHR saw a breach of Article 8 of the Convention. It stated that states are required to ensure recognition and protection of transsexual persons. Lithuania failed to legally regulate gender reassignment surgery thus keeping medical facilities from implementing opportunities for surgical treatment for transsexual individuals. Hence, according to the ECtHR, transsexual individuals in this country do not have the full legal acknowledgment of their correct gender and must live with a body not fully reassigned to the new gender.

#### 3.3.2. Judgments Involving Discrimination by Restriction of Parental Rights

Of the n = 7 cases concerning discrimination by restriction of parental rights, one (14.29%) concerned an individual with transsexuality (i), and 6 cases (85.71%) dealt with individuals with a different sexual orientation (ii).

Example of (i): The case A. M. and others v. Russia (appl. no. 47220/19) deals with a male-to-female transgender person who married her wife before undergoing her transition to a female sexual identity. The couple had two children. After getting a divorce, the applicant (the transgender person) was initially allowed to keep in touch with the children, but about one year later the former wife refused further visits from the applicant. During judicial proceedings, which were initiated by the former wife, an expert assessment came to the conclusion that visits of the applicant would put the children under considerable mental and psychosocial distress. As a result, the applicant was not allowed to have any contact with her children. In the opinion of the ECtHR, there was a violation of Article 8 and Article 14 of the Convention. The ECtHR considered the judgments of the domestic courts as most restrictive and being grounded on an insufficient and unbalanced examination of the entire situation of the family. Furthermore, the transsexuality of the applicant would disproportionately have been in the center of the domestic courts’ considerations.

Example of (ii): The case X and others v. Austria (appl. no. 19019/07) deals with a female same-sex couple. The two women lived unmarried together with the son of one of the partners. Having a stable relationship, they requested adoption of the child by the female same-sex partner. The domestic courts refused to approve the adoption agreement and dismissed subsequent appeals. The ECtHR’s judgment stated, in this case, a violation of Article 14 in conjunction with Article 8 of the Convention. The ECtHR argued that there was unequal treatment of the same-sex couple when compared with unmarried opposite-sex couples. Furthermore, the ECtHR constituted that, considerations on the concept of “traditional family”, which play an important part in Austrian jurisdiction, should inevitably be adapted to developments in society including new concepts of family.

#### 3.3.3. Judgments Involving Discrimination by the Disrespect of Sexual Identity or Sexual Orientation

We found n = 23 cases dealing with discrimination by the disrespect of sexual identity or sexual orientation. N = 16 of those (69.57%) concerned disrespect of sexual identity (i), n = 7 (30.43%) disrespect of sexual orientation (ii).

Example of (i): The applicant of the case B. v. France (appl. no. 13343/87) was a male-to-female transsexual of Algerian origin living in Paris. She underwent hormone therapy and gender reassignment surgery. In the course of her intention to marry her male partner, she requested to have her civil status register changed. This request was dismissed. It was argued that the applicant was not originally of female sex but intentionally changed her sex by artificial interventions not required by medical necessities. The ECtHR saw no reason why the civil status register could not be corrected, as this change merely would have been an actualization of the register by indicating the applicant’s correct gender. Moreover, the Court found that the frequent necessity to disclose the gender the applicant was born with as distressing in a degree which imposes a disrespect regarding her private life. The ECtHR identified a violation of Article 8 of the Convention.

Example of (ii): The applicant in Pajic v. Croatia (appl. no. 68453/13) was a woman seeking a residence permit in Croatia to live with her female partner. Her request and a subsequent constitutional complaint were dismissed; the authorities argued that the preconditions of a family reunification could not be met. In the opinion of the ECtHR, there was a violation of Article 14 in conjunction with Article 8 of the Convention. The Court argued that there was a difference of treatment in the case of the applicant that was neither objective nor reasonable. In the view of the Court, the jurisdiction in Croatia obviously is suffering from differences regarding the treatment of opposite-sex and same-sex relationships, which is not acceptable.

#### 3.3.4. Judgments Involving Discrimination by Jurisdiction

We identified a total of n = 67 cases (91.78%) in which discrimination of SGM individuals by the jurisdiction of the various states played a significant role. N = 16 of these cases (23.53%) dealt with persons of gender minority (i), n = 45 cases (67.16%) concerned persons of sexual minority (ii), and n = 7 cases (10.45%) concerned both individuals of gender and sexual minority.

Example of (i): In the case Christine Goodwin v. the United Kingdom (appl. no. 28957/95), the applicant, a post-operative male-to-female transsexual, complained about various situations in which she feels discriminated by the legal system on the grounds of her transsexualism. Even after her gender-reassignment surgery her National Insurance number, which must be revealed under certain circumstances, provided a way to trace back her former identity. Moreover, she was not able to get a state pension at the age of 60 which is the usual age to be eligible for State pension for women in the UK. In addition, she had to abstain from several advantages by not revealing her birth certificate stating her gender as male. In its considerations of this case, the Court stated that it is not apparent why the applicant on the one hand was able to profit from trans-specific medical interventions and on the other hand, was not continuously regarded as a female individual by jurisdiction. This situation would put the applicant in a tremendously stressful position making her vulnerable to humiliation. The Court stated that the chromosomal status of the applicant must not serve as the only parameter for assigning her gender, but that additional criteria must also be considered. In the opinion of the ECtHR, there was a breach of Article 8 of the Convention. Moreover, the Court also saw a violation of Article 12 of the Convention as the applicant was not legally able to marry a male person.

Example of (ii): The applicant in the case S. L. v. Austria (appl. no. 45330/99) was a male homosexual living in a rural area and suffering from stigmatization. Being sure of his homosexuality since age 15, he stated that he was not able to experience a fulfilling sexual relationship with an adult partner due to the legal prohibition of consensual sexual acts between adolescent and adult males. However, heterosexual and lesbian sexual acts between consenting adolescents over fourteen and adults were allowed. The ECtHR was of the opinion that there was a violation of Article 14 taken in conjunction with Article 8 of the Convention. It followed the argumentation of the applicant that it is not apparent why male homosexual adolescents in the age range between 14 to 18 should be protected against sexual relationships with adult men, while such protection should be unnecessary concerning young women and their potential sexual acts with either men or women.

#### 3.3.5. Judgments Involving Discrimination by the Prohibition of Promotion of Sexual Diversity

We found n = 6 judgments (8.22%) dealing with discrimination by prohibition of promotion of sexual diversity, including all SGM individuals.

Example: The case Genderdoc-M v. Moldova (appl. no. 9106/06) deals with a non-governmental LGBT organization seeking to arrange a demonstration against discrimination of sexual minorities. Authorization for the demonstration was denied and although the Court of Appeal judged in favor of the applicant in the first instance, it dismissed the applicant’s appeal after re-examination. The ECtHR stated that there was a violation of Article 11 of the Convention. Furthermore, it saw a breach of Article 13 in conjunction with Article 11: although there are regulations concerning the time-frame for decisions when a demonstration is announced, the authorities exceeded these time limits in a grotesque manner as the final decision on the request to hold the demonstration was delivered a year and a half after it had been lodged.

#### 3.3.6. Judgments Involving Discrimination by Verbal or Physical Attacks

We identified n = 12 cases (16.44%) involving discrimination by verbal or physical attacks. N = 5 cases (41.67%) concerned homosexual persons (i) and n = 7 cases (58.33%) concerned attacks against individuals of different sexual identity and orientation (ii).

Example of (i): The case Sabalic v. Croatia (appl. no. 50231/13) deals with a female homosexual suffering a physical attack during a visit to a nightclub after the attacker had been informed that she was homosexual. The applicant suffered several injuries which were qualified as minor bodily injuries during a physical examination. Although the police immediately responded at the scene, it only instituted minor offenses proceedings on the ground of a breach of public peace. A criminal and subsequent constitutional complaint of the applicant were both dismissed. The ECtHR saw a violation of Article 3 in conjunction with Article 14 of the Convention. In its argumentation, the Court assessed the injuries the applicant suffered could diminish her human dignity. The Court saw no doubt that the attacks had a homophobic motivation, and that this homophobia was not sufficiently addressed during the criminal proceedings.

An overview of all categories and sub-categories identified and the assigned judgments is provided in Table 3. Sub-categories are distinguishable topics that can be assigned to the respective main category.

## 4. Discussion

In our research, we have identified n = 73 ECtHR judgments pertaining to the issue of discrimination of SGM individuals in the Member States of the Council of Europe. Using the method of thematic analysis, we have categorized these judgments into six thematic categories. N = 7 cases (9.59%) concerned restricted access to healthcare, the rest of the cases (90.41%) other types of discrimination which were grouped into five categories. We grouped the cases in these categories regarding the main form of discrimination at hand. For instance, a restriction of parental rights in itself can surely be regarded as discrimination by jurisdiction but was separately discussed as we view it as a specific form of juridical discrimination.

The cases submitted to the ECtHR are only those that already have passed through all domestic remedies. They are by no means all of the cases debated in the Member States concerning specific topics and they represent just the tip of the iceberg. Thus, they can only be seen as an illustration of examples of discrimination and its consequences that SGM individuals face in their countries. However, the analyzed judgments show an important intersection between legal, medical, and ethical topics concerning sexual self-determination. They deal with topics such as access to healthcare services, legal and administrative discrimination, or social stigmatization of gay, lesbian, bisexual, transgender, and non-binary gender individuals. As such, judgments of the ECtHR can be regarded as normative texts on human rights violations. Their aim is to recognize violations of human rights and provide decisions ameliorating discriminative structures and actions in the Member States.

Remarkably, an adjustment of the cases to the legal situation regarding sexual orientation, reveals that 66.1% of cases concerning homosexual individuals come from countries in which non-heterosexual orientations are constitutionally or at least broadly protected, e.g., Sweden, Portugal [26]. A comparison with the legal situation of transgender individuals shows that 73.08% of all cases derive from states with a sufficiently developed legal system concerning transsexual persons and which do not have laws against transgender or gender diverse people, e.g., Austria, Germany [31]. A general climate of suppression, discrimination, and a lack of legal protection is clearly a significant hindrance for SGM individuals to claim their rights. As pointed out in the EU-LGBT II study, another reason for this is that SGM individuals fear that any legal dispute might not be successful at all and thereby they refrain from any legal proceedings [22].

Domestic laws abetting discrimination of SGM individuals put this vulnerable group at a risk to suffer from multiple negative biological and psychic outcomes. Experiences of discrimination (prejudice, internalized stigma, concealment of sexual minority status, etc.) relate to the negative impact on overall physical health, immune response, metabolic, hormonal, and cardiovascular parameters, and even higher risk of developing cancer [25]. By influencing domestic legal systems, the judgments of the ECtHR might help to save SGM individuals from discrimination and hence from a multitude of minority-stress-related health problems.

### 4.1. Cases Involving Limited Access to Healthcare

Most of the identified cases regarding a limitation of an access to healthcare concerned transsexual individuals and their unique medical needs pointing to a significant demand to improve their access to trans-specific treatment. Two major hindrances could be identified: a denial of the health insurance to cover the costs for the appropriate hormone and/or surgical treatment and legal uncertainties regarding the regulation of trans-specific care and its reimbursement. Without the possibility of receiving opposite-sex hormones and, if required and wished, gender reassignment surgery, transsexual persons suffer from gender dysphoria, where this can sufficiently be decreased by initiating appropriate treatment [32,33]. In its assessments, the ECtHR repeatedly points out that gender identification is protected by Article 8 of the Constitution as it falls under the broad term of “private sphere”. Furthermore, it regards the determination of one’s gender as an important part of one’s personal identity thus affecting a person’s freedom and dignity if this right to self-determination is violated. The lack of respect and support leads to transsexual persons being endangered to permanently suffer from distressing situations affecting their intimate life. It has been pointed out that the lack of access to healthcare providers that are sufficiently trained with respect to the specific needs of transsexual individuals is the single most barrier regarding access to healthcare for transsexual persons [7]. An abolishment of legal uncertainties and a reliable provision of reimbursement for trans-specific care seems to be a significant precondition for healthcare providers to engage in trans care and for medical education institutions to incorporate trans-specific knowledge in their curricula.

There is an ongoing debate on whether transsexualism should be regarded as a medical illness. We fully subscribe to the view that regarding transsexualism as a mental disease is a form of stigmatization that needs to be eliminated [34]. However, taking the cases discussed in this article into account, the acknowledgment of transsexualism as a disease on the other hand may serve as a guarantor for states to provide appropriate access to trans-specific care. One of the challenges of the future in trans-specific care will be enabling access to it without pathologizing transsexual persons as mentally ill. Some transsexual persons nowadays even feel relieved when “receiving” the necessary diagnosis [35]. In our opinion, the diagnosis of a mental illness should neither be the precondition for an acknowledgment of transsexualism nor be necessary for opening the gates to trans-specific care.

### 4.2. Cases Involving All Other Identified Types of Discrimination

Discrimination is a threat for SGM individuals. It puts them at a high risk to experience minority stress and thereby develop mental health problems such as depressive and/or anxiety symptoms [36]. Especially for transsexual individuals, it has been shown that they are at a greater risk for suicidality and non-suicidal self-injury [37]. A feeling of being victimized is associated with an increased probability to show suicidal behavior [38]. Furthermore, SGM individuals are at a greater risk to experience emotional distress as a consequence of experiencing bullying [39]. Thus, any form of discrimination can have a negative impact on the physical and mental health status of SGM individuals [16,17,18]. This might contribute to the persistent health disparities seen amongst the SGM population [40,41].

It is in line with these findings that the ECtHR in all analyzed judgments condemns any form of discrimination or violence on the grounds of sexual identity and/or sexual orientation. It frequently states that SGM individuals must be treated in the same way as the general population (e.g., S.L. v. Austria, appl. no. 45330/99) and that any failure to protect them from discrimination and/or violence or any failure to unmask possible discriminatory motives must be rectified (e.g., Identoba and others v. Georgia, appl. no. 73235/12). The ECtHR is aware of the possible negative impacts on SGM individuals’ health by noting that discriminatory acts may arouse feelings of fear, anguish or inferiority that may even break an individual’s moral or physical resistance (e.g., Sabalić v. Croatia, appl. no. 50231/13). The ECtHR also reacts to changes in the treatment of and attitude regarding SGM individuals by demanding different behavior of the Member States: concepts such as family or gender identity have to be re-thought and continuously reassessed in the light of new scientific insights and societal changes (e.g., X and others v. Austria, appl. No. 19010/07).

### 4.3. Limitations

There are several limitations to the presented study. The cases analyzed do not constitute all relevant cases regarding discrimination or limited access to healthcare which are disputed in the Member States but only a small proportion that has passed through all domestic instances. Therefore, the analyzed cases are not representative of all forms or prevalence of discrimination. However, the results of our investigation provide examples of discrimination that reach the EctHR and jurisprudence of the Court on these cases.

Furthermore, there are some limitations with respect to the method of our analysis. In our search, we included a broad number of terms that might be used to denominate SGM individuals. There still might be denominations that we missed. However, the majority of the relevant cases were found by using terms that might be relevant in a legal vocabulary (“homosexual”, “transsexual”, and “transgender”). We combined all these terms with the word “health”, as we specifically were interested in restricted access to healthcare. There might be more cases dealing with other forms of discrimination against SGM individuals. We do not claim to have been able to find every single judgment concerning every possible form of discrimination of SGM individuals. However, we are of the opinion that the results presented are sufficient to stress the relevant problem of discrimination of SGM individuals all over Europe. As described above, if a violation of one of the aspects respectively sub-paragraphs of an article of the EctHR was found, the respective article was counted as having been violated. Moreover, the articles in conjunction with which a violation of Article 14 was found, were counted as violated articles. A further differentiation as to which aspects of an article have been breached or whether an article has been found to be violated in conjunction with Article 14 or alone would have increased the complexity of the presented data without considerable additional value regarding the basic aim of our study. Moreover, researched and analyzed were only cases published in English. The database of EctHR includes also cases in other official languages; however, due to the language skills of the researchers and in order to ensure the reliability of the results, these cases were not included in the analysis.

The categories we used proved to be overlapping. It was difficult to clearly assign the cases to a certain category. Thus, we decided to assign the judgments to all those categories that most suitably were able to describe the case at hand. It cannot be excluded that some cases could have been assigned to yet another category or that the assignment to a certain category might be found to be disputable. Nonetheless, in our opinion, this did not impede the basic aim of this study to present the general outline of the respective EctHR’s assessments.

## 5. Conclusions

We found n = 73 judgments of the EctHR concerning a variety of discriminatory acts against SGM individuals (including restricted access to healthcare). In their assessments, the EctHR proves to have a balanced view on the sensitive topic of sexual self-determination. Most cases deal with discrimination by public authorities or the state. Discriminatory acts by individual persons are underrepresented in the cases analyzed. There is a need for further research regarding discrimination of SGM persons by other individuals, especially regarding the impact of this interpersonal discrimination on the restriction of access to healthcare, e.g., due to discrimination by healthcare providers. The data presented point to considerable discriminatory acts SGM individuals are experiencing all over Europe. Moreover, their access to healthcare, especially the access of transsexual individuals to transition-specific interventions, can be restricted. Being aware of these circumstances may help clinicians to interact with this vulnerable group more sensitively and to explore their specific needs more adequately. Awareness that SGM individuals might have a history of discriminatory experiences and restrictions regarding their access to healthcare can help healthcare professionals to better understand their specific situation and to reduce any barriers that might arise for SGM individuals in healthcare. To provide more open-minded atmospheres, healthcare professionals should, if needed, be supported to develop a positive and affirming attitude towards SGM individuals. This could, by way of example, be achieved by specific training or incorporation of relevant information regarding SGM individuals in the curricula of medical schools and universities.

## Figures and Tables

**Table 1:** Frequencies of articles on rights and freedom (Section 1, Articles 1–18) and protocols of the European Convention on Human Rights in the n = 70 judgments included in this analysis.

Articles of the European Convention on Human Rights	Name of the Article	Judgments Involving This Article	Judgments in Which at Least One Violation of This Article Was Found (Either Alone or in Conjunction with Other Articles)
Article 3	Prohibition of torture	n = 11 (15.07%)	n = 8 (10.96%)
Article 5	Right to liberty and security	n = 3 (4.11%)	n = 3 (4.11%)
Article 6	Right to a fair trial	n = 6 (8.22%)	n = 4 (5.48%)
Article 8	Right to respect for private and family life	n = 55 (75.34%)	n = 42 (57.53%)
Article 9	Freedom of thought, conscience and religion	n = 1 (1.37%)	n = 0
Article 10	Freedom of expression	n = 6 (8.22%)	n = 2 (2.74%)
Article 11	Freedom of assembly and association	n = 9 (12.33%)	n = 8 (10.96%)
Article 12	Right to marry	n = 9 (12.33%)	n = 2 (2.74%)
Article 13	Right to an effective remedy	n = 10 (13.70%)	n = 6 (8.22%)
Article 14	Prohibition of discrimination	n = 57 (78.08%)	n = 33 (45.21%)
Article 18	Limitation on use of restrictions on rights	n = 1 (1.37%)	n = 1 (1.37%)
Article 1 of Protocol No 1	Protection of property	n = 1 (1.37%)	n = 1 (1.37%)
Article 1 of Protocol No 12	General prohibition of discrimination	n = 1 (1.37%)	n = 0

**Table 2:** Number of identified judgements and violated Articles of the Convention.

Country	No. of Judgments	Violation of …										
		Art. 3	Art. 5	Art. 6	Art. 8	Art. 10	Art. 11	Art. 12	Art. 13	Art. 14	Art. 18	Art. 1 of Protocol 1
United Kingdom	15				8			2	1	1		1
Austria	13				11	1			1	10		
Russia	8		2	2	1	1	5		2	6	1	
France	5			1	3							
Italy	4				4					1		
Poland	3				2					2		
Croatia	2	1			1					2		
Georgia	2	2					1	1		2		
Lithuania	2	2					2			2		
Moldova	2	1					1		1	2		
Portugal	2	1			1					1		
Romania	2				1		1			2		
Turkey	2	1			1					1		
Bulgaria	1											
Cyprus	1				1							
Finland	1											
Germany	1											
Greece	1				1					1		
Hungary	1		1									
Ireland	1				1							
Macedonia	1				1							
Spain	1											
Sweden	1											
Switzerland	1		1									

**Table 3:** Overview of the categories and sub-categories identified and the assigned judgments.

Main Category	Number (Percentage) of Judgments in the Main Category	Sub-Categories	Number (Percentage) of Judgments in Subcategories	Examples Presented in the Text
Limited access to healthcare	n = 7 (9.59%)	Restriction of reimbursement of hormone therapy and/or gender reassignment therapy	n = 1 (14.29%)	Van Kück v. Germany (35968/97)
		Difficulty to receive hormone therapy and/or gender reassignment surgery due to legal uncertainties	n = 3 (42.86%)	L. v. Lithuania (27527/03)
		Denial of artificial insemination	n = 1 (14.29%)	
		Denial of extension of health insurance	n = 1 (14.29%)	
		Denial of access to medical doctor	n = 1 (14.29%)	
Restriction of parental rights	n = 7 (9.59%)	Concerning transsexual individual	n = 1 (14.29%)	
		Concerning homosexual individuals	n = 6 (85.71%)	X and others v. Austria (19019/07
Disrespect of sexual identity/orientation	n = 23 (31.51%)	Concerning disrespect of sexual identity	n = 16 (69.57%)	B. v. France (13343/87)
		Concerning disrespect of sexual orientation	n = 7 (30.43%)	Pajic v. Croatia (68453/13)
Discrimination by jurisdiction	n = 67 (91.78%)	Concerning transsexual individuals	n = 15 (22.39%)	Christine Goodwin v. the United Kingdom (28957/95)
		Concerning homosexual individuals	n = 45 (67.16%)	S. L. v. Austria (45330/99)
		Concerning trans- and homosexual individuals	n = 7 (10.45%)	
Prohibition of promotion of sexual diversity	n = 6 (8.22%)			Genderdoc-M v. Moldova (9106/06)
Verbal or physical attacks	n = 12 (16.44%)	Concerning homosexual individuals	n = 5 (44.44%)	Sabalic v. Croatia (50231/13)
		Concerning trans- and homosexual individuals	n = 7 (55.56%)	

## Data Availability

Data for this research are publicly available through the database HUDOC of the European Court of Human Rights, accessible under https://hudoc.echr.coe.int/eng#{%22documentcollectionid2%22:[%22GRANDCHAMBER%22,%22CHAMBER%22]}. Last access: 4 February 2022.
